# Fetal and Maternal Outcomes in Women With Major Placenta Previa Among Sudanese Women: A Prospective Cross-Sectional Study

**DOI:** 10.7759/cureus.14467

**Published:** 2021-04-13

**Authors:** Mohamed Alkhatim Alsammani, Khalid Nasralla

**Affiliations:** 1 Department of Obstetrics and Gynecology, College of Medicine, Qassim University, Buraidah, SAU; 2 Department of Obstetrics and Gynecology, College of Medicine, University of Bahri, Khartoum, SDN

**Keywords:** placenta previa, maternal complications, fetal complications, prevalence rate, sudan khartoum

## Abstract

Background

Placenta previa is a major obstetric problem with high rates of fetomaternal mortality and morbidity. This study aimed to determine the prevalence and fetal and maternal outcomes of major degree placenta previa among Sudanese women.

Method

This is a prospective descriptive study conducted in the period from January 1 to June 30, 2109, at Omdurman Maternity Hospital, Khartoum, Sudan. Fetal and maternal complications associated with major degree placenta were analyzed using descriptive statistics.

Results

The total number of deliveries was 22,000, of which 87 cases were of major degree placenta previa, giving a prevalence rate of 0.4%, the hysterectomies rate was 23% (n= 20), and the total maternal deaths were 6.9% (n= 6). Intraoperative interventions used to control the bleeding were multiple hemostatic sutures in 34.5% (n=30) of cases, followed by uterine backing (20.7%; n= 18), and uterine artery ligation (12.6%; n=11). The common reported maternal complications were bladder injuries (28.7%; n= 25) followed by bowel injuries (4.6%; n=5). Of all mothers, 48.27% (n=42) were admitted to the intensive care unit (ICU). Of all deliveries, 26.4% (n=23) were preterm, and 38% (n=33) of neonates were admitted to the newborn intensive care unit (NICU), and 9.2% (n=8) were fresh stillbirth (FSB).

Conclusion

Neonatal complications were comparable to other studies but maternal deaths were relatively high. The study indicated the need for effective management protocols and more training of the medical staff in order to overcome the problem.

## Introduction

Placenta previa has a worldwide prevalence of 0.3%-0.5%, and it constitutes a major obstetric dilemma, with a high risk of fetomaternal mortality and morbidity [1]. Women with placenta previa were usually old, multiparous, had a (1) prior cesarean section, in addition, some may have had a history of the morbidly adherent placenta [1-3]. Other risk factors of placenta previa include infertility treatment, multiple gestations, smoking, male fetus, and black race [1,4].

The most common and fatal complication of placenta previa is massive obstetric hemorrhage, and increased morbidity is mainly due to blood transfusion, hysterectomy, and surgical injuries to the bladder, bowel, or ureter [2].

Babies born to mothers with placenta previa have a 15% chance of delivery before 34 weeks gestation. This puts the baby at risk for complications related to prematurity such as respiratory distress syndrome, growth restriction, low birth weight, and increased risk of cerebral palsy and hypoxic-ischemic encephalopathy [4].

Hemorrhage of placenta previa is no longer a major cause of maternal mortality in developed countries due to the availability of protocols and well-equipped health facilities [5], which is not the scenario in the low-income sub-Saharan Africa where obstetric hemorrhage is still a leading cause of fetomaternal mortality and morbidity [6].

Sudan is one of those countries in which hemorrhage due to placenta previa still constitutes a major health challenge. The aim of this study is to determine the prevalence, frequency of complications, and methods of surgical intervention among mothers suffering from major degrees of placenta previa in Khartoum, Sudan.

## Materials and methods

Study design and setting

A prospective, cross-sectional hospital-based study was conducted to investigate the prevalence and complications among women with major degree placenta previa This study was carried in the period from January 1 to June 30, 2109, in Omdurman Maternity Hospital, Khartoum, Sudan. Omdurman Maternity Hospital is considered a referral center receiving patients from all parts of the Sudanese capital Khartoum. The policy of the hospital includes giving dexamethasone 12 mg I/M, two doses 12 hours apart for all patients diagnosed with placenta previa before 34 weeks gestation; also, all patients with placenta previa should have cross-matched blood ready before surgery.

Data collection

A structural questionnaire was used to gather the data by the trained registrar in obstetrics and gynecology.

Definitions

The diagnosis of major placenta previa was based on ultrasound or intraoperative findings. In our settings, MRI is not used as a secondary tool to diagnose placenta previa because it is not available at our hospital.

Questionnaire

The questionnaire included gathering data on maternal and fetal complications along with other demographic data, including age, parity, occupation, education, weight, height, and residence. The recorded maternal complications include: hemorrhage, anemia, blood transfusion, wound infection, hysterectomy, injury to bowel, bladder, or ureter, ICU admission, and death. The fetal complications reported are prematurity, NICU admission, and fetal death.

Blood loss is estimated by the total weight of abdominal gauzes used and subtracting the known weight of the same materials when dry. The difference in weight in grams approximates the volume of blood in milliliters.

Statistics analysis

The Statistical Package for Social Sciences (SPSS) version 24 (IBM Corp., Armonk, NY) descriptive statistics was used for recording and analyzing data.

Ethical considerations

Oral informed consent was obtained from all participants in the study. The study was reviewed & approved by the hospital ethics committee and the ethical committee of the Sudan Medical Specialization Board, and all patients consented to a possible hysterectomy if it became a life-saving procedure.

## Results

The total number of deliveries during the study period was 22,000 deliveries of which there were 87 cases of placenta previa, thus the prevalence of placenta previa among our population was 0.4%. The mean age group, height, weight, body mass index, and parity were: 32.83 ± 3.35 years, 161.94 ± 6.68 cm, 84.2 ± 14.76 kg, 32.0018 ± 4.63, and 2.08 ± 4.7 deliveries, respectively. The mean number of cesarean sections was 3.04 ± 1.46.

The majority of our women population were college graduates (55.2%; n=48) and the minority were illiterate (8% n=7). Almost half of the population were employees (44.8%, n=39) as compared to 43.7% (n=38) who were housewives. Seventy-nine point thirty-five percent (79.35%; n=69) of the population were from urban areas. Thirty-three point three percent (33.3%; n=29) of women had a previous history of placenta previa and only 2.3% (n=2) had a history of dilatation and curettage (D&C) (Table 1).

**Table 1:** Clinical and demographic data of women with placenta previa GA: gestational age

GA	36.9500±5.77507	
Number of CS.	3.0357±1.45988	
Parity	2.0814±.46565	
Education		
Illiterate	7(8)	
Primary	12(13.8)	
Secondary	19(21.8)	
University	48(55.2)	
Occupation		
Housewife	38(43.7)	
Employer	39(44.8)	
Other	9(10.3)	
Residence		
Urban	69(79.3)	
Rural	18(20.7	
Previous uterine surgery	2(2.3)	
Past history	29(33.3)	

Nine point two percent (9.2%; n=8) of all women did not require any intraoperative surgical intervention while the majority of cases who required intervention had responded to multiple uterine sutures (34.5%; n=30). Uterine backing, uterine artery ligation, and hysterectomy were performed in 20.7% (n=18), 12.6% (n=11), and 23% (n=20), respectively (Table 2).

**Table 2:** Intraoperative intervention among women with placenta previa

No intervention	8 (9.2)
Multiple sutures	30 (34.5)
Uterine backing	18 (20.7)
Baking+ uterine artery ligation	11(12.6)
Hysterectomy	20 (23)

The mean gestational age at delivery, the average fetal weight, and the mean Apgar score at five minutes were 36.95 ± 5.77 weeks, 3.09 ± 0.288 kg, and 8.27 ± 1.53, respectively. Of all babies, the premature deliveries were 26.4% (n=23), 9.2% (n=8) were fresh stillbirths, and 38% (n=33) of them required NICU admission (Table 3).

**Table 3:** Neonatal outcomes among women with placenta previa NICU: newborn intensive care unit; FSB: fresh stillbirth

Gestational age	36.9500±5.77507
Fetal weight at delivery	3.0923±.28823
Gender	
Male	41(43.6)
Female	39(41.5)
NICU	
Yes	33(38)
NO	54(62)
Macrosomia	2(2.3)
Normal weight	56(64.4)
Preterm babies	23(26.4)
Fetal death	
Alive	79(90.8)
FSB	8(9.2)
Apgar score at 5 min	8.2727±1.52726

The most common reported maternal complication in the present study was intraoperative bleeding (71.3%; n=67). Forty-eight point twenty-seven percent (48.27%; n=42) required ICU admission. Bowel and bladder injuries were reported in 4.6% (n=5) and 28.7% (n=25), respectively. Of those who were hemodynamically unstable, 52.87% (n=46) received a blood transfusion.

The observed postpartum complications included secondary postpartum hemorrhage (PPH), endometritis, pulmonary embolism, and anemia, which were 4.6% (n=5), 2.3% (n=2), 21.8% (n=19), and 25.3% (n=22), respectively. The reported maternal deaths were 6.9% (n=6). Wound infections occurred in 10.4% (n=9) cases. The average units of blood transfused and the time of hospital stay were 3.5 ± 1.8 units and 9.9 ± 3.8 days, respectively (Table 4).

**Table 4:** Maternal outcomes among women with placenta previa ICU: intensive care unit: PPH: postpartum hemorrhage; APH: antepartum hemorrhage

Bowel injuries	5 (4.6)
Bladder injuries	25 (28.7)
ICU	42 (48.27)
2^nd^ PPH	5 (4.6)
Endometritis	2 (2.3)
Re-laparotomy	5 (4.6)
Pulmonary embolism	19 (21.8)
Maternal death	6 (6.9)
APH	29 (33.3)
Anemia	22 (25.3)
Transfusion	46 (52.87)
Intraoperative diagnosis	19 (21.8)
Intraoperative bleeding	67 (71.3)
Maternal death	
Yes	6 (6.9)
No	81 (93.1)

## Discussion

The estimated overall prevalence of placenta previa ranges from 0.4% to 0.52% [7-8]. In a large systemic review, the incidence of placenta previa was highest among Asians (1.2%) and lowest in sub-Saharan Africans (0.27%) [7]. Another retrospective cohort study also showed that Asians have the highest rate of placenta previa (0.64%) and the lowest rates were among African Americans and Hispanics - 0.44% and 0.34%, respectively. In a regional descriptive study, the reported prevalence of placenta previa is 1.3% [2], which was even higher than the above-stated prevalence for Asians (1.2%) [7]. The prevalence of placenta previa in this study is 0.4%, which is comparable to that of the African Americans and less than the reported regional North African prevalence, which may support the idea of ethnic variation with lower rates among the black race as compared to Asians. Ethnicity was suggested as one of the causes for this regional variation in the prevalence rate of PP.

The risks of developing placenta previa are many, including maternal age ˃ 35 years, gravidity of 3 or more, parity of 2 or more, and history of previous cesarean deliveries [3,9]. The sociodemographic factors in our study population, namely, the means of age, parity, and the number of previous cesarean deliveries were 32.8±3.4, 2.1±0.5, and 3±1.5, respectively, and 33.3% of our population had a past history of placenta previa. All these factors are comparable to the known risk factors reported in the literature [3,9].

It is known that placenta previa is associated with adverse fetomaternal outcomes [10]. Maternal mortality and antepartum hemorrhage (APH) are the most catastrophic outcomes in those patients. The risk of APH in women with placenta previa is nine times more than in those with the normally situated placenta [6], and the reported rate is 51.6% [11]. Obviously, the 33.3% prevalence of APH in this study is less than the above-mentioned rate possibly due to the sample size in these studies and the difficulty to qualify blood loss due to massive obstetrics hemorrhage because of the lack of a uniform method to assess blood loss.

Maternal mortality has regional variation, but, generally, it ranges from 5%-7% [12] and in spite of the fact that maternal mortality in sub-Saharan Africa accounts for two-thirds of the global maternal death rate, there was a significant reduction in the maternal mortality ratio (MMR) in north Africa and other parts of the world in the period from 2000 to 2017 [13]. A regional North African descriptive study, which involved 52 cases of major degree placenta previa, reported no maternal deaths and in another cross-sectional study conducted in West Africa, the maternal death rate was as high as 15.6% [14].

The death rate among our study population is 6.9%, which is considered high if compared regionally with the North African countries or globally with the high-income countries but, still, this rate is comparable to the rates in other parts of Africa. This high maternal death rate may be partially due to a delay in decision-making to proceed for cesarean hysterectomy in cases of bleeding, which reflects the urgent need for the formulation of effective management protocols.

Cesarean hysterectomies were performed in 23% of our study population, which is considered relatively high if compared to the reported rate of cesarean hysterectomy due to placenta previa in a large population-based comparative study, which was 5.3% Vs 0.04% in women with the normally situated placenta (10). Also, our cesarean hysterectomy rate is higher than the locally reported rate (15.1%) [2] but, still, it is comparable to the rate reported from the South African cross-sectional study (21.5%) [15].

Data from South Africa showed that the common intraoperative interventions needed to control massive bleeding were uterine artery ligation, uterine backing using an intrauterine balloon, and a B-Lynch suture; the rate of each was: 7.5%, 4.3%, and 16.1%, respectively [15]. In our study, the procedures performed were multiple hemostatic sutures in 34.5%, uterine backing in 20.7%, and uterine artery ligation in 12.6% of cases. The internal iliac ligation was not performed in any of our participants, which may be due to its technical difficulty and ineffectiveness of internal iliac ligation [16], also, the B-Lynch suture and its modified form were not used in spite of their easiness and effectiveness as recommended in several studies, which may be partly due to a lack of skill to perform these procedures [17-18].

The bowel injuries (4.6%) and bladder injuries (28.7%) recorded in this study were high when compared to the reported rates in the literature, which is 0.44% for bladder injuries [19] and 0.08% for bowel injuries [20]. These rates are still high if compared to regional rates (3.8% for bowel injuries and 13.2% for bladder injuries [2]. These high rates may reflect the need for more training programs for both junior and senior medical staff.

The reported risk of postpartum wound infections is 5% [21] and the regional risk is 17.3% [2] while our rate of wound infection is 10.4%, which is almost better than the reported regional rate. The prevalence of endometritis in this study is 2.3%, which is less than the reported prevalence of 3.8%-8.7% [22].

Perinatal complications increase in cases of placenta previa. A tertiary center study conducted in Mumbai, India, has reported that the rates of NICU admissions and FSB in patients with placenta previa were 18% and 3.3%, respectively [23]. Recorded rates from regional data were 17.3% for NICU admissions [2], 13.2% [2], and 4.16% [24] for FSB rate. Our FSB rate was 9.2% and NICU admission rate was 38%, both comparable with later regional studies.

Preterm birth is 14 folds higher in women with placenta previa [9]. In a systematic review, the preterm birth rate in women with placenta previa was 57.7% [25] and the rate in another Israeli study was 52% as compared with the only 8% preterm birth rate in patients with normally situated placenta [26]. The rate of preterm birth among our study population was only 26.4%, which is far less than the above-mentioned rates but, still, it is comparable and even more than the the13.5% rate that was reported regionally [2].

## Conclusions

The prevalence of placenta previa in our study is comparable to the prevalence among African Americans and less than that reported for Asians, which may support the ethnic variation in the prevalence of placenta previa with higher rates in blacks. The relatively high maternal deaths may be partially solved by introducing effective management protocols and avoiding any possible delay in decision-making when a cesarean hysterectomy is needed. Also, the high rate of intraoperative surgical injuries may reflect the need for more training programs that involve both senior and junior medical staff.
